# Capturing COVID-19Like Symptoms at Scale Using Banner Ads on an Online News Platform: Pilot Survey Study

**DOI:** 10.2196/24742

**Published:** 2021-05-20

**Authors:** Brian E Dixon, Sumit Mukherjee, Ashley Wiensch, Mary L Gray, Juan M Lavista Ferres, Shaun J Grannis

**Affiliations:** 1 Department of Epidemiology Richard M Fairbanks School of Public Health Indiana University Indianapolis, IN United States; 2 Center for Biomedical Informatics Regenstrief Institute Indianapolis, IN United States; 3 AI for Good Research Lab Microsoft Corporation Redmond, WA United States; 4 New England Lab Microsoft Research Cambridge, MA United States; 5 Luddy School of Informatics, Computing, and Engineering Indiana University Bloomington, IN United States; 6 Department of Family Medicine Indiana University School of Medicine Indianapolis, IN United States

**Keywords:** COVID-19, coronavirus, epidemiology, research subject recruitment, signs and symptoms

## Abstract

**Background:**

Identifying new COVID-19 cases is challenging. Not every suspected case undergoes testing, because testing kits and other equipment are limited in many parts of the world. Yet populations increasingly use the internet to manage both home and work life during the pandemic, giving researchers mediated connections to millions of people sheltering in place.

**Objective:**

The goal of this study was to assess the feasibility of using an online news platform to recruit volunteers willing to report COVID-19like symptoms and behaviors.

**Methods:**

An online epidemiologic survey captured COVID-19related symptoms and behaviors from individuals recruited through banner ads offered through Microsoft News. Respondents indicated whether they were experiencing symptoms, whether they received COVID-19 testing, and whether they traveled outside of their local area.

**Results:**

A total of 87,322 respondents completed the survey across a 3-week span at the end of April 2020, with 54.3% of the responses from the United States and 32.0% from Japan. Of the total respondents, 19,631 (22.3%) reported at least one symptom associated with COVID-19. Nearly two-fifths of these respondents (39.1%) reported more than one COVID-19like symptom. Individuals who reported being tested for COVID-19 were significantly more likely to report symptoms (47.7% vs 21.5%; *P*<.001). Symptom reporting rates positively correlated with per capita COVID-19 testing rates (*R^2^*=0.26; *P*<.001). Respondents were geographically diverse, with all states and most ZIP Codes represented. More than half of the respondents from both countries were older than 50 years of age.

**Conclusions:**

News platforms can be used to quickly recruit study participants, enabling collection of infectious disease symptoms at scale and with populations that are older than those found through social media platforms. Such platforms could enable epidemiologists and researchers to quickly assess trends in emerging infections potentially before at-risk populations present to clinics and hospitals for testing and/or treatment.

## Introduction

The global outbreak of SARS-CoV-2 led the World Health Organization (WHO) to declare a pandemic on March 11, 2020 [1]. SARS-CoV-2 causes COVID-19, which has a range of manifestations from asymptomatic infection to severe pneumonia, potentially leading to intensive care utilization and death. With over 34 million cases globally, COVID-19 has impacted health and health care in every country. Although COVID-19 spread has leveled off in some parts of the world [2], public health experts anticipate future outbreaks given that only a fraction of the population has been infected with the disease [3].

Given the need to isolate or quarantine infected individuals in order to slow community-level spread, a key component of an effective response to COVID-19 is early identification of new cases. At the beginning of the pandemic, most nations lacked capacity to confirm positive cases, typically performed using a real-time reverse transcriptionpolymerase chain reaction laboratory test. Over time, public health as well as hospital and commercial labs have expanded their abilities to perform COVID-19 tests. However, COVID-19 is challenging to identify because many cases are mild or asymptomatic. Between 40% and 45% of active viral cases do not exhibit symptoms [3,4]. Other individuals experience more mild symptoms, such as muscle aches, that may not prompt them to seek medical care. Individuals who do not feel sick are less likely to present to hospital or clinic for testing. Therefore, many COVID-19 cases go unreported, making it challenging for epidemiologists to track COVID-19 spread or model its future impact.

There exist several infodemiology [5,6] and digital surveillance tools to identify or estimate suspected COVID-19 cases in a population. These tools seek to capture COVID-19 symptoms, risks, and exposure information directly from consumers. Several recent studies report efforts to capture COVID-19 symptom data from consumers using internet-based surveys [7,8] or social media platforms [9,10]. Additional studies examine the use of mobile apps that allow patients to self-report symptoms; some are dedicated to COVID-19, while others are extensions of existing platforms [11-13]. Further, some studies explore the use of web search behaviors to detect potential clusters of COVID-19 [14].

Existing COVID-19 surveillance tools tend to focus on tracking COVID-19 cases in a localized geographic area. For example, a review of COVID-19 mobile apps found that more than half were official apps managed by local health authorities presumably focused in their local region [13]. Moreover, many reports on existing tools either lack details regarding users who provided information [12,14] or the population surveyed was skewed, such as in Shen et al [9] where 85% of respondents were between 19 and 40 years of age. To maximize efficacy, digital public health surveillance tools should capture data from a representative sample across local, state, and national levels to enable global outbreak identification and trend monitoring. Furthermore, public health authorities should use a variety of surveillance tools to gather data from multiple sources to triangulate disease outbreaks and trends.

In this study, we sought to examine the feasibility of using an existing web-based news platform to recruit individuals willing to self-report symptoms associated with COVID-19 as well as health behaviors via a survey. We sought to recruit a diverse set of volunteers in a manner that can be scaled during a pandemic.

## Methods

### Overview

We employed an epidemiologic survey to collect COVID-19 symptoms and behaviors from individuals recruited using a web-based news platform. Not knowing whether the method would be successful, we approached the project as a feasibility study. We collected data in multiple countries over 4 weeks to examine the platforms ability to capture a representative sample of the underlying population with internet access in order to detect suspected COVID-19 cases before individuals may have sought diagnosis and treatment from the health system.

### Survey Instrument

The survey primarily focused on capturing symptoms associated with COVID-19. Using the best available data at the time (early April 2020), the following symptoms were included in the survey: fever, cough, itchy or watery eyes, loss of sense of taste or smell, nasal congestion or runny nose, sore throat, and shortness of breath or difficulty breathing. Our list was principally based on the list published by the US Centers for Disease Control and Prevention, which recognized three main symptoms for COVID-19: fever, cough, and shortness of breath [15]. An early systematic review, published in March 2020, that included 1576 early COVID-19 patients reported that the most prevalent clinical symptom was fever, followed by cough, fatigue, and dyspnea [16]. A later review reported the main symptoms to be fever, cough, fatigue, slight dyspnea, sore throat, headache, conjunctivitis, and gastrointestinal issues [17]. We further included anosmia (loss of the sense of smell) and ageusia (loss of the sense of taste), because early evidence [18] linking these symptoms was emerging from the literature around the time of survey development. Because these symptoms were only associated with COVID-19, and they are similar to symptoms experienced by patients with other diseases, we considered these symptoms to indicate COVID-19like illness rather than a definitive case of COVID-19, which is confirmed through laboratory testing.

In addition to symptoms, we asked respondents to report some behaviors linked to COVID-19 risk. We specifically asked respondents to describe where they had spent time outside of their home in the past 2 weeks, more than 18 miles from home or fewer than 18 miles from home. We also asked if they had traveled outside of their home country in the previous 2 weeks.

We collected minimal demographics for respondents to protect privacy. The only demographics requested were age group (eg, 40-49 years) and gender. We further asked for respondents postal codes (eg, ZIP Code) to enable comparisons with localized outbreaks of COVID-19. We did not ask respondents to separately report their country of residence. We did not capture race data as it is not customary to collect such information outside the United States.

We created the survey in English and subsequently translated it into 12 additional languages for release into 30 different language markets. Languages included, among others, Mandarin, French, German, Japanese, Greek, and Spanish.

### Subject Recruitment

To recruit participants, we placed banner ads on the Microsoft News platform. Microsoft News delivers news from multiple, popular publishers across web and mobile experiences. The service is used by nearly a half billion monthly users, making it one of the largest audiences of news readers in the world [19].

The Microsoft News team embedded a link to the Regenstrief Institute survey on its news sites in 31 global language markets to reach a large, general population sample for the survey. Each language market directed users to a survey in that language. Initially, Microsoft News editors manually embedded a promotion button calling for survey participants that linked to the survey. Editors randomly embedded the link in approximately 150 pieces of news content daily that were delivered to readers across the United States. Editors placed a button on news content at randomized time windows (eg, 5:36 AM Pacific Time [PT]; 2:12 PM PT) to limit the time window in an effort to deter malicious tampering (eg, bot attacks) during a 1-week survey pilot period. Unique URLs for each language-specific survey were also used to prevent bot attacks. Editors also attempted to avoid placing the button on COVID-19related content to prevent oversampling people reading about COVID-19. Moreover, editors tried to ensure the button was placed at a low profile (ie, below the fold of the news article).

By April 10, 2020, COVID-19related content accounted for 80% of US news traffic. This challenged the manual embedding process, placing an unmanageable burden on the Microsoft News editorial team. Therefore, in consultation with the research team, Microsoft News switched to a conditional banner method on April 11, 2020.

**Figure 1:**
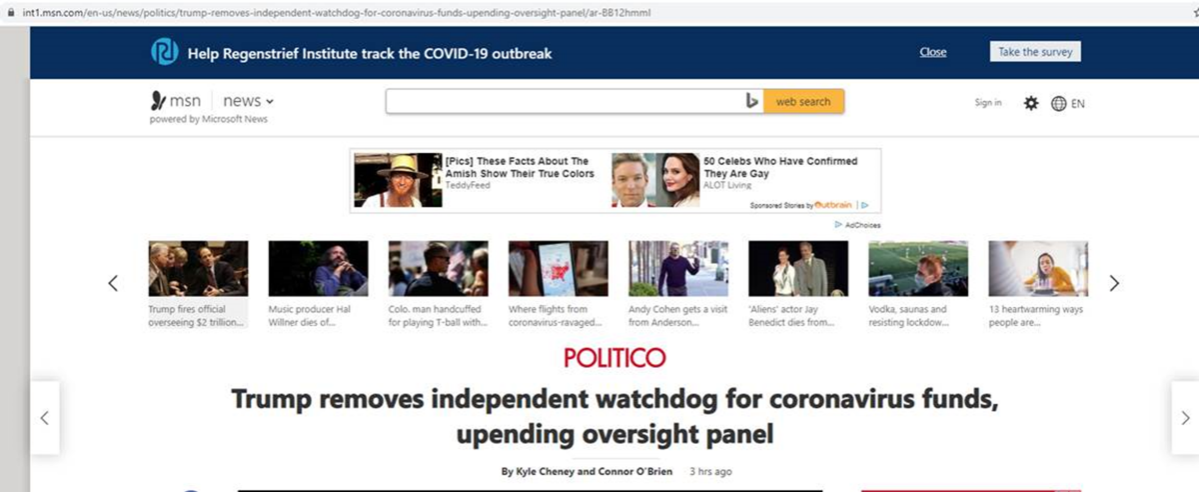
Screenshot of the Microsoft News platform showing the banner ad placed at the top of the screen that recruited participants to take a survey about COVID-19 symptoms and behaviors.

The conditional banner ad methodology involved automatically rendering the ad (Figure 1) at the top of all news articles, but with the condition that a user would only see the banner twice in 30 days. The conditional banner provided a gating effect so that Microsoft News readers could only be served a total of two conditional banners calling for participants to complete the survey during any single month. For example, if a reader visited a travel article and a politics article during the month, the reader had two opportunities to see the banner ad but would not see it again until the next month. Furthermore, once a reader clicked on a conditional banner ad, which took them to the survey, they would not receive another conditional banner ad calling for participants until the next month. We offered users two banner ads per month until they completed the survey or until the survey closed. Although Microsoft News tracked whether a user saw the banner ad more than once, user identifiers were not provided to the research team, so we could not identify individuals or link their multiple responses.

Importantly, Microsoft News readers who clicked on the banner ad always saw an initial screen on the survey site that prompted them for their explicit consent to participate, informed them of the risks of participating, and gave them a chance to leave the survey and either return to Microsoft News or access COVID-19 information resources (eg, WHO site). No user data were captured until participants consented. The Regenstrief Institute monitored referring websites to confirm that nearly all respondents came to the survey from Microsoft News.

### Data Collection

**Figure 2:**
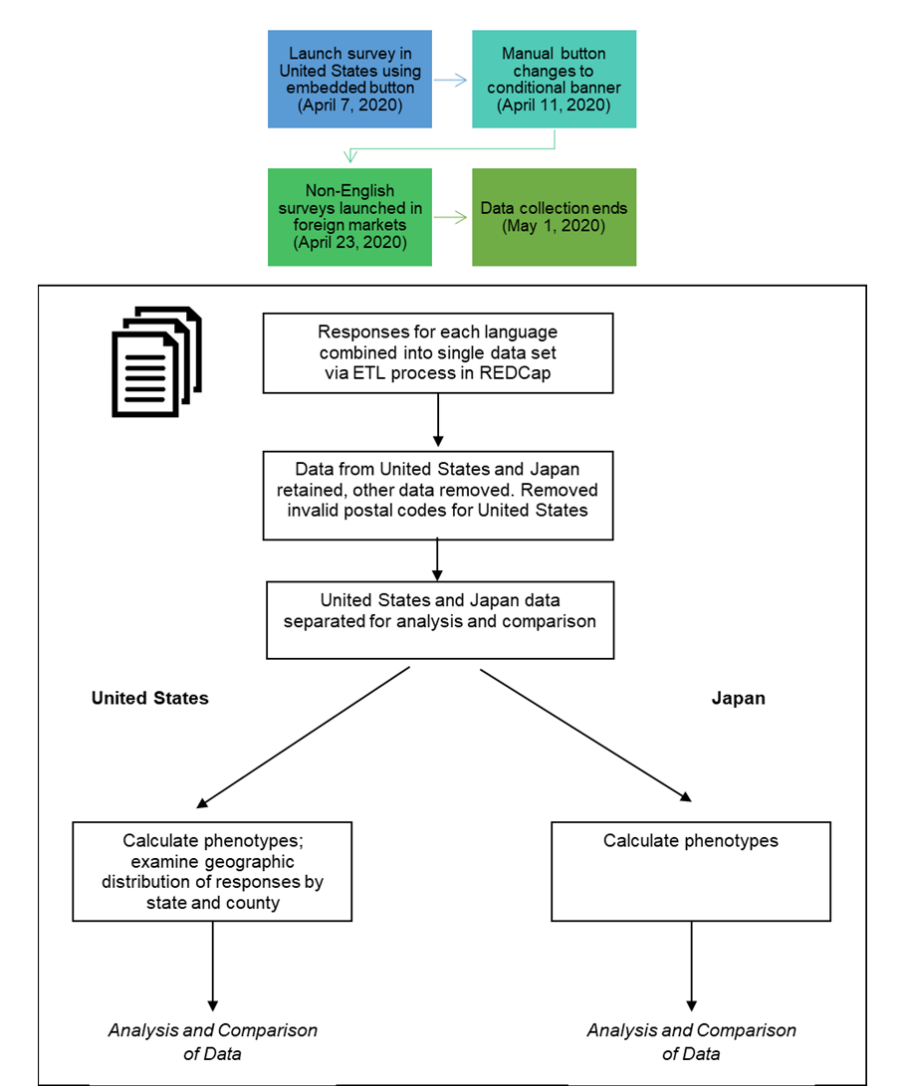
Overview of data collection and analysis processes. ETL: extract, transform, load; REDCap: Research Electronic Data Capture.

Surveys were developed and rolled out in two phases. We first generated and published the US-based English survey. The US-based English survey was published beginning on April 7, 2020. Beginning April 11, 2020, the conditional banner ad replaced the manually embedded button. Non-English surveys were published on April 23, 2020. All surveys closed on May 1, 2020. Figure 2 depicts the overall flow for collection and analysis of data.

Survey responses were collected and managed in collaboration with Research Technologies, a Pervasive Technology Institute Center at Indiana University (IU) [20], using Research Electronic Data Capture (REDCap) tools [21,22]. Data from each language-based survey were stored in separate tables on the REDCap server, then extracted into a combined data set for analysis using REDCap-ETL (extract, transform, load) and the IU REDCap-ETL Gateway [23,24]. The language of the respondent was captured as a distinct field in the combined data set.

REDCap is a secure, web-based software platform designed to support data capture for research studies, providing (1) an intuitive interface for validated data capture, (2) audit trails for tracking data manipulation and export procedures, (3) automated export procedures for seamless data downloads to common statistical packages, and (4) procedures for data integration and interoperability with external sources. REDCap-ETL extracts data from REDCap, transforms the extracted data, and loads the transformed data into a database. The IU REDCap-ETL Gateway enables high-throughput data transfer between REDCap and multiple endpoints by allowing REDCap-ETL to scale to many simultaneous runs using the job scheduler of a high-performance computer.

### Analytical Methods

We used descriptive statistics to summarize responses overall and by country to examine patterns. We further employed ecological methods to compare prevalence by country as well as association methods to examine differences in responses by country. Moreover, we employed correlation methods to compare symptom responses to standard outbreak measures. More than 85% of responses were received from residents in the United States and Japan. Therefore, we only included these responses in the analysis.

We first examined patterns regarding the limited demographics captured in the survey. Descriptive statistics provide a sense for the representativeness of the respondents. We calculated summary statistics for respondent demographics after removing all entries that did not provide informed consent, indicated by failing to click *Next*. In all demographic categories, we restricted our summarization to those individuals who did not select *prefer not to say*.

We next summarized responses based on respondents reported symptoms and behaviors that might contribute to COVID-19 exposure. We asked respondents to indicate the presence of one or more current symptoms, including symptoms believed to be associated with COVID-19 infection and those not shown to be associated. The symptoms believed to be more common among patients testing positive for COVID-19 include fever, cough, shortness of breath, loss of sense of taste or smell, and sore throat [7,17,18,25].

Symptoms associated with COVID-19 were grouped into various phenotypes for COVID-19like illness. We defined four different COVID-19 phenotypes for analysis, summarized in Table 1. We examined a variety of phenotypes to explore symptom combinations associated with COVID-19. We examined multiple phenotypes since evidence suggests that many COVID-19 patients experience more than one symptom, but a specific set of symptoms is not yet definitive. Phenotype 3 is the broadest definition for a potential case, as individuals could report any of the symptoms previously known to be associated with COVID-19 infection. Phenotype 4 is the most restrictive phenotype, as it requires indication of fever or loss of taste or smell plus one of the other symptoms associated with infection. All phenotypes were informed by available evidence [26] indicating which symptoms or combinations are associated with infection.

**Table 1:** Phenotypes for suspected COVID-19 cases.

Symptoms	Phenotype 1	Phenotype 2 (one or the other)		Phenotype 3	Phenotype 4 (both must apply)	
	Individual reports *at least* 2 of the following	Individual reports *any* 1 of the following	Individual reports *at least* 2 of the following	Individual reports *any* 1 of the following	Individual reports *at least* 1 of the following	Individual reports *any* 1 of the following
Fever						
Cough						
Loss of sense of taste or smell						
Sore throat						
Shortness of breath or difficulty breathing						

To visualize the distribution of respondents in the United States, we restricted the data to valid US ZIP Codes. The text field used by respondents to enter their postal code was unrestricted, given the lack of global conventions for representing residential area. Invalid ZIP Code entries were ignored when generating maps of US respondents. States were assigned based on published ZIP Code values associated with each state. To map ZIP Code values to US counties, we leveraged a crosswalk between county Federal Information Processing System codes and ZIP Code.

Chi-square tests of independence were used to examine whether responses differed significantly between the United States and Japan. We further compared responses between respondents indicating that they received testing versus those reporting that they did not receive laboratory testing for COVID-19. We employed correlation to compare the proportion of respondents meeting various phenotype definitions in a given state to existing surveillance per capita metrics reported in the same state during the study period. First, we correlated phenotypes with the per capita testing rate in each state, since individuals with symptoms are theoretically more likely to seek out testing. Second, we correlated phenotypes with the reported per capita case rate, which represents the positive case volume in a given state. These metrics were obtained from The Atlantic application programming interface [27], which are aggregated from a variety of sources.

### Ethics Approval

This study was approved by the Institutional Review Board at IU as exempt research.

## Results

We received a total of 87,322 valid responses. Of these, 47,424 (54.3%) were from the United States and 27,936 (32.0%) were from Japan. Table 2 summarizes responses and the demographics of respondents. Responses from Japan are skewed toward male gender (21,220/27,936, 77.0%), whereas the US responses are more balanced (male: 25,787/47,424, 57.0%). With respect to age, more than half of the respondents from both countries were over 50 years of age. Chi-square analysis found the rates of responses in Japan were significantly different from those in the United States.

**Figure 3:**
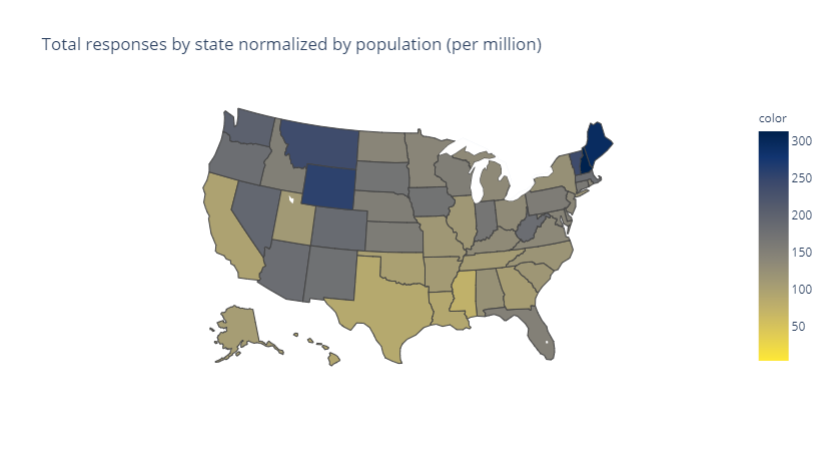
Total responses by US state normalized by population. Lighter colors indicate low counts, whereas darker colors indicate a higher number of responses.

The distribution of respondents in the United States is depicted in Figure 3 as well as in Multimedia Appendix 1. The raw counts of respondents from each state and ZIP Code (Figures S1 and S2 in Multimedia Appendix 1) show broad geographical distribution of responses across the nation. Higher numbers of responses were generally observed in areas with higher populations. When normalized by state population, responses were greatest in the northern states, especially the extreme northeastern states and the states in the Upper Plains region.

**Table 2:** Demographics of all respondents (who completed the consent portion) of a web-based questionnaire about COVID-19 symptoms recruited through the Microsoft News platform.

Demographic			All respondents, n (%)^a^		Respondents from the United States, n (%)^a^		Respondents from Japan, n (%)^a^		*P* value^b^
**Age (years) (all: n=83,901; United States: n=45,050; Japan: n=27,536)**									
	80+	2672 (3.2)		2202 (4.9)		252 (0.9)		<.001	
	70-79	12,150 (14.5)		8402 (18.7)		2343 (8.5)		<.001	
	60-69	23,632 (28.2)		14,438 (32.0)		6233 (22.6)		<.001	
	50-59	23,174 (27.6)		10,635 (23.6)		9539 (34.6)		<.001	
	40-49	12,580 (15.0)		5040 (11.2)		5850 (21.2)		<.001	
	30-39	5208 (6.2)		2567 (5.7)		1757 (6.4)		<.001	
	18-29	4485 (5.3)		1766 (3.9)		1562 (5.7)		<.001	
**Gender (all: n=84,134; United States: n=45,195; Japan: n=27,461)**									
	Female	29,171 (34.7)		19,221 (42.5)		6174 (22.5)		<.001	
	Male	54,651 (65.0)		25,787 (57.1)		21,220 (77.3)		<.001	
	Transgender	180 (0.2)		102 (0.2)		40 (0.1)		<.001	
	Nonbinary	132 (0.2)		85 (0.2)		27 (0.1)		<.001	

^a^Percentages may not add up to 100% due to rounding.

^b^*P* values were based on chi-square analysis.

**Figure 4:**
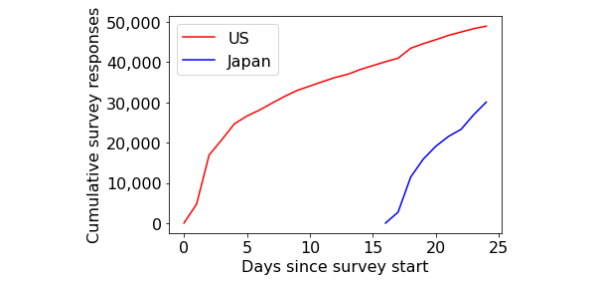
Cumulative responses over time, stratified by location of respondent, to a survey on COVID-19 symptoms. The survey was offered in Japan 16 days after it was introduced in the United States.

Responses over time are summarized in Figure 4, stratified by the location of the response. Cumulative responses increased rapidly in the days immediately after the survey launch followed by a continued, steady increase in the weeks after the launch. The Japanese survey was launched 16 days after the English survey, yet responses followed a similar pattern with respect to rapid increase following the launch.

In Table 3 we summarize the responses with respect to COVID-19 symptoms, phenotypes, and behaviors. Most respondents (56,551/87,322, 64.8%) reported no symptoms. Among those reporting at least one symptom (30,771/87,322, 35.2%), a total of 19,631 respondents (63.8%) reported at least one of the following symptoms associated with COVID-19: fever, cough, loss of sense of taste or smell, sore throat, shortness of breath, or difficulty breathing. These respondents met the inclusion criteria for Phenotype 3. Higher proportions of respondents in the United States reported COVID-19like symptoms than respondents in Japan across all symptoms and phenotypes (42.2% vs 25.2%; *P*<.001).

Among the 19,631 respondents meeting criteria for Phenotype 3, 7670 (39.1%) reported at least two symptoms associated with COVID-19 (Phenotype 1). A total of 15,341 out of 30,771 individuals with symptoms (49.9%) reported either cough or shortness of breath, or they reported at least two symptoms from the set of fever, sore throat, or loss of taste or smell (Phenotype 2). Examining the more specific symptoms of fever and loss of sense of taste or smell, 4820 out of 30,771 individuals with symptoms (15.7%) reported at least one of these symptoms in combination with one of the other symptoms (Phenotype 4). Reported symptoms met phenotype definitions at higher rates among respondents from the United States compared to respondents from Japan (*P*<.001).

Compared to respondents who did not report symptoms, those reporting at least one of the symptoms associated with COVID-19 (Phenotype 3) in the United States were more likely to report getting tested (*P*<.001). Similar rates were observed in Japan. Overall, higher proportions of respondents in the United States reported getting tested than did those in Japan (*P*<.001). In both countries, individuals meeting the inclusion criteria for one of the phenotypes more frequently reported that they had traveled more than 30 km from their home within the past 14 days. Chi-square analysis found that the rates of responses in Japan were significantly different from those in the United States.

**Table 3:** Symptoms, phenotypes, and behaviors reported by respondents using a web-based questionnaire recruited through the Microsoft News platform.

Symptoms, phenotypes, and behaviors			All respondents (N=87,322), n (%)		Respondents in the United States (n=47,424), n (%)		Respondents in Japan (n=27,936), n (%)		*P* value^a^
**Individual symptom**									
	Fever	3308 (3.8)		2428 (5.1)		527 (1.9)		<.001	
	Loss of taste or smell	2585 (3.0)		2032 (4.3)		214 (0.8)		<.001	
	Cough	12,826 (14.7)		9189 (19.4)		2184 (7.8)		<.001	
	Shortness of breath	5768 (6.6)		4502 (9.5)		495 (1.8)		<.001	
	Sore throat	8021 (9.2)		5135 (10.8)		1873 (6.7)		<.001	
	Runny nose	17,157 (19.6)		11,772 (24.8)		3567 (12.8)		<.001	
	Itchy or watery eyes	12,800 (14.7)		8866 (18.7)		2518 (9.0)		<.001	
	None	56,551 (64.8)		27,411 (57.8)		20,888 (74.8)		<.001	
	At least 1 symptom	30,771 (35.2)		20,013 (42.2)		7048 (25.2)		<.001	
**Phenotype**									
	Phenotype 1	7670 (8.7)		5837 (12.2)		924 (3.3)		<.001	
	Phenotype 2	15,341 (17.4)		10,961 (23.0)		2498 (8.9)		<.001	
	Phenotype 3	19,631 (22.3)		13,308 (27.9)		3881 (13.8)		<.001	
	Phenotype 4	4820 (5.5)		3647 (7.6)		623 (2.2)		<.001	
**Tested**									
	Phenotype 1	635 (8.3)		551 (9.4)		23 (2.5)		<.001	
	Phenotype 2	827 (5.4)		707 (6.5)		32 (1.3)		<.001	
	Phenotype 3	939 (4.8)		793 (6.0)		45 (1.2)		<.001	
	Phenotype 4	523 (10.9)		461 (12.6)		21 (3.4)		<.001	
	None	869 (1.5)		496 (1.8)		227 (1.1)		<.001	
**Traveled more than 30 km**									
	Phenotype 1	1539 (20.1)		1242 (21.3)		164 (17.7)		<.001	
	Phenotype 2	2724 (17.8)		2052 (18.7)		436 (17.5)		<.001	
	Phenotype 3	3394 (17.3)		2433 (18.3)		659 (17.0)		<.001	
	Phenotype 4	994 (20.6)		799 (21.9)		107 (17.2)		<.001	
	None	9237 (16.3)		5070 (18.5)		3181 (15.2)		<.001	

^a^*P* values were based on chi-square analysis.

In Table 4, we compare respondents who reported that they were tested for COVID-19 versus those who reported no testing. Overall, few respondents (1967/87,322, 2.3%) indicated that they were tested for COVID-19. Examining symptom combinations and phenotypes revealed that individuals who reported symptoms were more likely to report being tested for COVID-19. Among those tested for COVID-19, nearly half (939/1967, 47.7%) reported at least one symptom compared to individuals who were not tested (18,197/84,498, 21.5%; *P*<.001). The most prevalent symptom combinations among individuals tested for COVID-19 included cough and nasal congestion or runny nose (404/1967, 20.5%), cough and shortness of breath or difficulty breathing (372/1967, 18.9%), as well as fever and cough (318/1967, 16.2%). Nearly two-thirds (55,084/84,498, 65.2%) of individuals who reported no symptoms also reported not being tested.

The proportion of respondents in each state meeting a given phenotype definition positively correlated with the proportion of individuals tested in that state (Figures S1-S4 in Multimedia Appendix 2). For example, the proportion of individuals who reported symptoms corresponding to Phenotype 3 were significantly (*P*<.001) positively (*R*^2^=0.26) correlated with the per capita rate of individuals tested for COVID-19 (Figure S3 in Multimedia Appendix 2). The proportion of respondents meeting a given phenotype definition showed slight correlation with the reported case rate (*R*^2^0.04) (Figures S5-S8 in Multimedia Appendix 2), yet these correlations were not statistically significant (*P*.05). For example, the proportion of individuals who reported symptoms corresponding to Phenotype 4 were nonsignificantly (*P*=.23) positively (*R*^2^=0.04) correlated with the per capita rate of individuals testing positive for COVID-19 (Figure S8 in Multimedia Appendix 2).

**Table 4:** Symptom combinations and phenotypes among respondents, stratified by whether or not they reported getting tested for COVID-19.

Symptom combinations and phenotypes		All respondents (N=87,322), n (%)	Respondents tested for COVID-19 (n=1967), n (%)	Respondents not tested for COVID-19 (n=84,498), n (%)	*P* value^a^
**Individual symptom combination**					
	Fever and cough	2268 (2.4)	318 (16.2)	1895 (2.2)	<.001
	Fever and itchy or watery eyes	1345 (1.4)	146 (7.4)	1162 (1.4)	<.001
	Fever and loss of sense of taste or smell	1073 (1.1)	189 (9.6)	850 (1.0)	<.001
	Fever and nasal congestion or runny nose	1821 (1.9)	227 (11.5)	1548 (1.8)	<.001
	Fever and sore throat	1666 (1.8)	205 (10.4)	1411 (1.7)	<.001
	Fever and shortness of breath or difficulty breathing	1626 (1.7)	254 (12.9)	1327 (1.6)	<.001
	Cough and itchy or watery eyes	4713 (5.0)	246 (12.5)	4338 (5.1)	<.001
	Cough and loss of sense of taste or smell	1625 (1.7)	244 (12.4)	1327 (1.6)	<.001
	Cough and nasal congestion or runny nose	6523 (6.9)	404 (20.5)	5963 (7.1)	<.001
	Cough and sore throat	4056 (4.3)	310 (15.8)	3654 (4.3)	<.001
	Cough and shortness of breath or difficulty breathing	3650 (3.9)	372 (18.9)	3186 (3.8)	<.001
	Itchy or watery eyes and loss of sense of taste or smell	1228 (1.3)	129 (6.6)	1056 (1.2)	<.001
	Itchy or watery eyes and nasal congestion or runny nose	7382 (7.8)	271 (13.8)	6932 (8.2)	<.001
	Itchy or watery eyes and sore throat	3166 (3.4)	173 (8.8)	2921 (3.5)	<.001
	Itchy or watery eyes and shortness of breath or difficulty breathing	2430 (2.6)	173 (8.8)	2179 (2.6)	<.001
	Loss of sense of taste or smell and nasal congestion or runny nose	1585 (1.7)	209 (10.6)	1324 (1.6)	<.001
	Loss of sense of taste or smell and sore throat	1147 (1.2)	162 (8.2)	945 (1.1)	<.001
	Loss of sense of taste or smell and shortness of breath or difficulty breathing	1241 (1.3)	194 (9.9)	1000 (1.2)	<.001
	Nasal congestion or runny nose and sore throat	4079 (4.3)	250 (12.7)	3729 (4.4)	<.001
	Nasal congestion or runny nose and shortness of breath or difficulty breathing	3125 (3.3)	272 (13.8)	2765 (3.3)	<.001
	Sore throat and shortness of breath or difficulty breathing	2112 (2.2)	226 (11.5)	1826 (2.2)	<.001
**Phenotype**					
	Phenotype 1	7670 (8.1)	635 (32.3)	6857 (8.1)	<.001
	Phenotype 2	15,341 (16.3)	827 (42.0)	14,117 (16.7)	<.001
	Phenotype 3	19,631 (20.8)	939 (47.7)	18,197 (21.5)	<.001
	Phenotype 4	4820 (5.1)	523 (26.6)	4166 (4.9)	<.001
	No symptoms	56,551 (60.0)	869 (44.2)	55,084 (65.2)	<.001

^a^*P* values were based on chi-square analysis.

## Discussion

### Principal Findings

In this study, we recruited tens of thousands of respondents within 3 weeks to an online survey asking questions about symptoms associated with a novel infectious disease quickly spreading across the globe using banner ads on an online news platform. A principal finding is that an internet news platform is feasible for quickly collecting large-scale data from individuals across local, state, and national areas. Furthermore, this recruitment method reached an older population at greater risk of complications from COVID-19, distinguishing our study from prior research. Whereas many infodemiology studies recruit subjects from social media platforms where users are primarily under 40 years of age, the news platform we leveraged draws primarily from populations at least 50 years of age. Both findings have implications for future surveillance efforts in public health.

**Figure 5:**
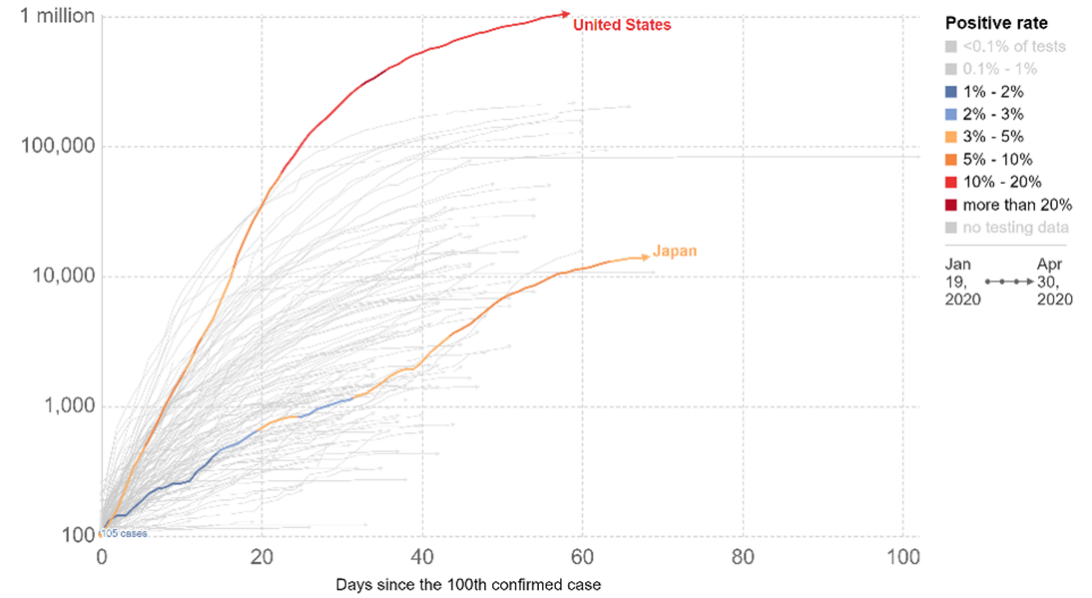
COVID-19 epidemic curves for the United States and Japan through May 1, 2020 [26]. The plot shows cumulative confirmed COVID-19 cases. The number of confirmed cases is lower than the number of actual cases; the main reason for that is limited testing. The source of this data is the European Centre for Disease Prevention and Control, Situation Update Worldwide, last updated August 31, 2020, 10:34 AM (London time). Official data were collated by Our World in Data [28].

At time of the survey, Japans epidemic curve had started to flatten, with daily increases in new cases between 3% and 5%, whereas the curve in the United States continued its upward trajectory, with daily increases between 10% and 20% (Figure 5 [26,28]). On April 30, 2020, the United States reported a daily rate of new infections of 8.26 per 100,000 population. On the same date, Japan reported a daily rate of 0.19 new infections per 100,000 population. In this study, rates of reported symptoms in Japan were significantly lower than in the United States, providing face validity for respondents self-reported data. This further builds the case for an online news platform as a reliable method for recruiting survey respondents.

The survey data further yielded interesting insights about the pattern of symptoms reported by respondents. First, nearly two-thirds of those reporting at least one symptom reported at least two symptoms. Emerging evidence suggests that symptom combinations may be predictive of COVID-19 infection [26]. The fact that most individuals reporting COVID-19like symptoms reported more than one symptom provides further evidence that exploring specific phenotypes might be fruitful for future research. Second, the constellation of symptoms involving loss of taste or smell with fever appears more often among individuals who reported that they received testing for COVID-19. Several studies report loss of taste and smell as common among COVID-19 patients [18,26,29], suggesting that these symptoms cause individuals to seek out medical care or indicate an association with infection. Moreover, those reporting at least one symptom were significantly more likely to report having been tested for COVID-19 than those without symptoms, and those reporting symptoms were significantly associated with testing rates. Yet the rates of individuals reporting symptoms were not significantly associated with positive case rates, which were admittedly hindered due to limited testing infrastructure in the United States at the time of the survey [30]. These findings are novel, given the data were captured from community residents rather than individuals hospitalized from COVID-19, which is the population most prior studies used to report common symptoms reported by individuals infected with SARS-CoV-2.

### Implications of Findings

Based on these findings, we encourage further study of online news platforms as a tool for reaching broad and diverse populations by public health agencies during emerging outbreaks. Although the news platform was male dominated and skewed older with respect to population, we recommend its use in combination with other epidemiologic methods, including infodemiology, random selection, and contact tracing. Only by using multiple methods will public health organizations successfully achieve diverse representation in the total set of data used to inform disease tracking efforts. Surveying only young, healthy populations is not sufficient for ascertaining prevalence of diseases like COVID-19. Yet inclusion of younger population data is important to understand disease spread during a crisis. It is possible that other news platforms may have younger audiences or cater to specific audiences that would improve diversity. Capturing data across platforms and methods to integrate findings would benefit future infodemiology research.

The methods used in this study go beyond the typical methods used in public health to ascertain symptoms among individuals who present at clinical or public health sites. Relying on data from individuals who present physically for treatment or testing alone is not sufficient to generate population-level information about the epidemiology of an emerging infectious disease, as only some individuals present for care or testing. Recall that approximately 40% of individuals with COVID-19 are asymptomatic [3], and many experience mild symptoms that do not require clinical intervention. Moreover, we received responses from individuals in small towns and rural communities, areas that are often medically underserved, thereby limiting patients ability to present for care. Furthermore, rural areas often lack robust laboratory testing infrastructures, a broad challenge that COVID-19 illuminated. Finally, the methods used here allowed for at-scale collection of data in a way that respects individuals privacy, as respondents did not need to provide any data that might otherwise be used to link back to medical records or sensitive information.

Internet-based survey methods are needed to gather data quickly during a crisis to inform where the disease might be present or where it might be headed next. Modeling efforts at the start of the COVID-19 pandemic were limited by a paucity of input data as well as a priori assumptions that drive model parameters. Having intelligence on symptoms from a broad population as well as behaviors (eg, those travelling >30 km from home) could better inform model development. Moreover, understanding which populations have received testing, and which ones do not appear to have access to testing, can inform deployment of test kits by national and/or state public health agencies. Finally, identification of symptom hot spots could help drive deployment of personal protective equipment as well as health care workers.

### Limitations and Future Directions

Despite the promise of the methods described in this study, there remain many challenges for using internet news platforms and other infodemiology methods to inform public health action. Given limited testing infrastructure in many states, analysis of survey data in comparison with later waves of the pandemic might better show how survey data correlate with standard epidemiologic metrics, such as testing rates, case rates, and mortality. Furthermore, the descriptive nature of this study prevented examination of potential sampling bias in respondents. We observed some skewing related to gender and age. Further exploration of the methods for ad placement would be necessary to establish news platforms as a reliable method for infodemiology.

The study was further limited by the location data being restricted to postal codes. Postal code structures are not standardized globally. Some postal codes repeat in the world, which prevented accurate mapping of some responses to a correct country or province. Furthermore, some entries in the postal code field were unintelligible. It is also possible that some of the Japanese responses occurred outside Japan, as postal code information was self-reported rather than verified with location services within the web browser or mobile device. Moreover, the survey did not ask whether those tested for SARS-CoV-2 virus tested positive or negative. Therefore, we considered individuals reporting symptoms to have a COVID-19like illness, recognizing that COVID-19 symptoms are similar to several respiratory diseases, such as influenza. The symptom list was further restricted to the best available evidence at the time of survey development.

In the future, we plan to expand our work to extend the survey to additional audiences and in combination with other methods for reaching broader audiences. We further plan to update the symptom list and capture additional details about an individual and their health history that could be correlated with COVID-19 cases and/or known risk factors for infection as well as hospitalization and death. In addition, because severely ill patients may not be able to complete an online survey, we will also explore whether our survey might be adapted to allow respondents to report information on family members who may be experiencing COVID-19like symptoms.

### Conclusions

News platforms can be used to quickly recruit study participants, enabling collection of infectious disease symptoms at scale and with populations that are distinct from those found through social media platforms. Such platforms could enable epidemiologists and researchers to quickly assess trends in emerging infections potentially before individuals present to clinics and hospitals for testing and/or treatment.

## Appendix

Multimedia Appendix 1 Distribution of responses by US state and county.

Multimedia Appendix 2 Correlations of individuals meeting phenotype definitions with existing surveillance metrics.

## Abbreviations

ETL: extract, transform, load
IU: Indiana University
PT: Pacific Time
REDCap: Research Electronic Data Capture
WHO: World Health Organization
