# Supplementary material for: Capturing COVID-19Like Symptoms at Scale Using Banner Ads on an Online News Platform: Pilot Survey Study
Source: J Med Internet Res. 2021 May 20;23(5):e24742. doi: 10.2196/24742 (PMC8139394; doi:10.2196/24742)

Multimedia Appendix 1

Distribution of responses by U.S. state and county

Dixon et al., JMIR #24742

Figure S1. Total counts of responses by U.S. state. Lighter colors indicate low counts, whereas darker colors indicate a higher number of responses.
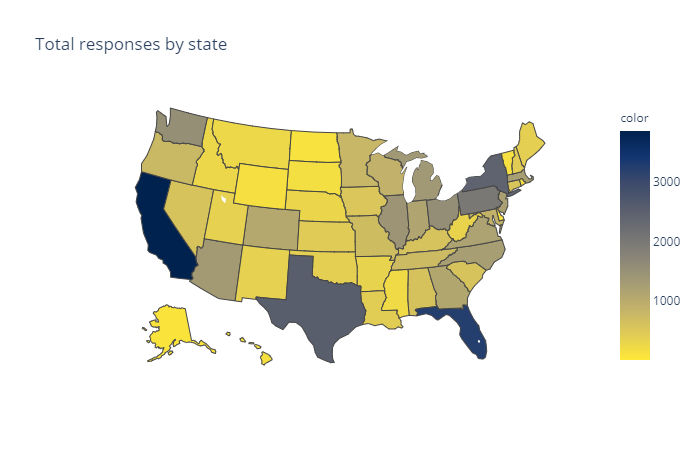


Figure S2. Count of responses in U.S. counties. Lighter colors indicate low counts, whereas darker colors indicate a higher number of responses.


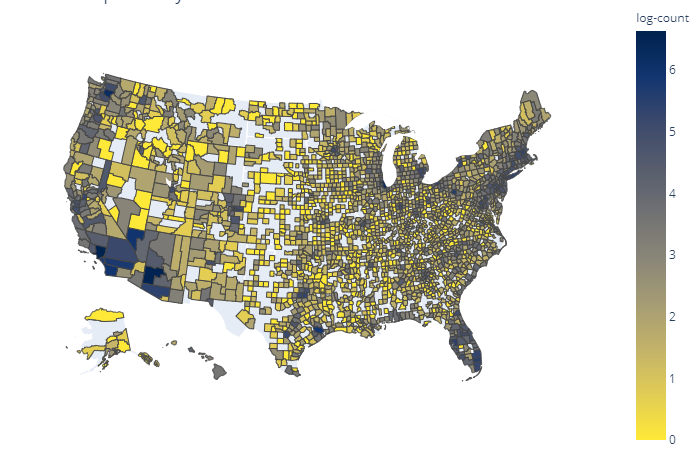

Supplement: Multimedia Appendix 1 [file jmir_v23i5e24742_app1.docx]
