# Supplementary material for: Capturing COVID-19Like Symptoms at Scale Using Banner Ads on an Online News Platform: Pilot Survey Study
Source: J Med Internet Res. 2021 May 20;23(5):e24742. doi: 10.2196/24742 (PMC8139394; doi:10.2196/24742)

Multimedia Appendix 2

Correlations of individuals meeting phenotype definitions with existing surveillance metrics.

Dixon et al., JMIR #24742

**Correlation of phenotypes with testing rates per capita**

Figure S1. The proportion of respondents meeting criteria for Phenotype 1 in a given state correlated with the testing rate of the same state per capita.


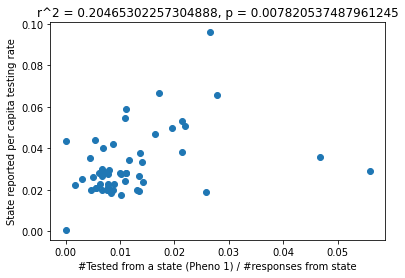


Figure S2. The proportion of respondents meeting criteria for Phenotype 2 in a given state correlated with the testing rate of the same state per capita.


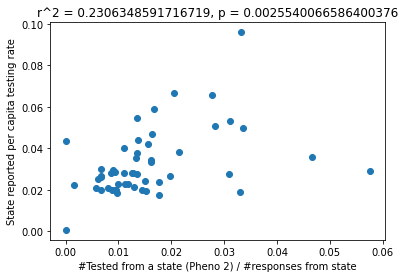


Figure S3. The proportion of respondents meeting criteria for Phenotype 3 in a given state correlated with the testing rate of the same state per capita.


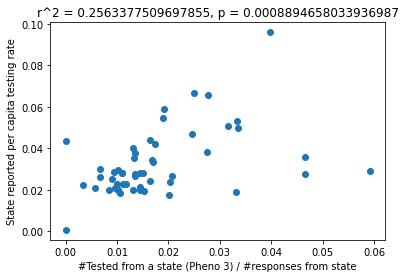


Figure S4. The proportion of respondents meeting criteria for Phenotype 4 in a given state correlated with the testing rate of the same state per capita.


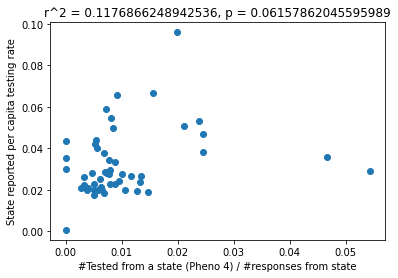


**Correlation of phenotypes with reported cases per capita**

Figure S5. The proportion of respondents meeting criteria for Phenotype 1 in a given state correlated with the per capita reported case rate for the same state.


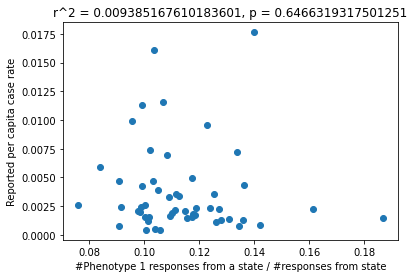


Figure S6. The proportion of respondents meeting criteria for Phenotype 2 in a given state correlated with the per capita reported case rate for the same state.


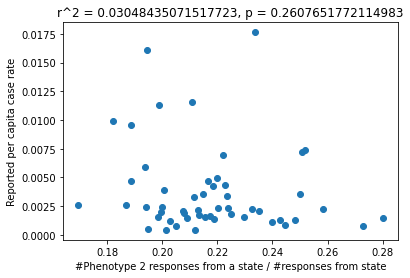


Figure S7. The proportion of respondents meeting criteria for Phenotype 3 in a given state correlated with the per capita reported case rate for the same state.


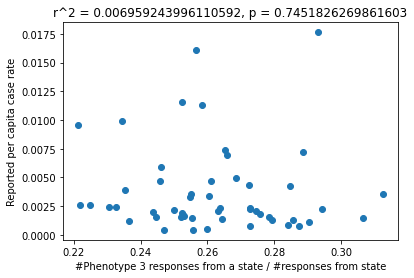


Figure S8. The proportion of respondents meeting criteria for Phenotype 4 in a given state correlated with the per capita reported case rate for the same state.


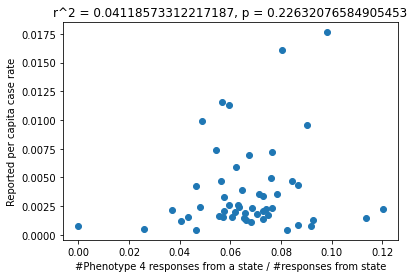

Supplement: Multimedia Appendix 2 [file jmir_v23i5e24742_app2.docx]
